# Predicting High Risk for Human Hantavirus Infections, Sweden

**DOI:** 10.3201/eid1501.080502

**Published:** 2009-01

**Authors:** Gert E. Olsson, Marika Hjertqvist, Åke Lundkvist, Birger Hörnfeldt

**Affiliations:** Swedish Institute for Infectious Disease Control, Solna, Sweden (G.E. Olsson M. Hjertqvist, Å. Lundkvist); Swedish University of Agricultural Sciences, Umeå, Sweden (G.E. Olsson); Swedish Defence Research Agency, Umeå (G.E. Olsson); Karolinska Institutet, Stockholm, Sweden (Å. Lundkvist); Umeå University, Umeå (B. Hörnfeldt)

**Keywords:** Hantavirus, rodents, predicting, outbreak, temporal pattern, Sweden, dispatch

## Abstract

An increased risk for hemorrhagic fever with renal syndrome caused by Puumala hantavirus was forecast for Sweden in 2007. The forecast was based on a predicted increase in the number of *Myodes glareolus* rodents (reservoir hosts). Despite raised awareness and preparedness, the number of human cases during July 2007–June 2008 was 1,483, a new high.

Puumala virus (PUUV) is the etiologic agent of nephropathia epidemica, a mild form of hemorrhagic fever with renal syndrome (HFRS). PUUV is likely to be the most prevalent hantavirus in Europe (*1*); the bank vole *Myodes* (*Clethrionomys*) *glareolus*, one of the most widespread and abundant mammal species on the continent, is a natural reservoir (*2*–*4*). PUUV is excreted from bank voles by saliva, urine, and feces, and transmission to humans often occurs by inhalation of aerosolized excreta. An average of 10–40 HFRS cases are serologically confirmed per 100,000 population every calendar year in HFRS-endemic northern Sweden (*5*,*6*). However, in 2007 an all-time high of 2,195 cases were reported in Sweden, almost 4 times the previous record (n = 564 in 1998). A total of 807 cases, or 313 cases/100,000 population, were reported from Västerbotten County, a HFRS-endemic area. Approximately 90% of all HFRS cases are found within the HFRS-endemic northern region of Sweden (*5*), but cases outside this region most often originate from the HFRS-endemic areas and are found during the summer season as a consequence of residents spending their holidays in the north (*7*). The all-time high in HFRS diagnoses during the winter of 2006–2007 may have been affected not only by high bank vole numbers but also indirectly by extreme winter conditions (*7*).

We monitored data on small mammals from northern Sweden and used these data to predict and subsequently verify high bank vole numbers in the fall of 2007, which indicated an increased risk of PUUV exposure to humans. We discuss additional factors that may alter bank vole–human encounter rates and influence the occurrence of HFRS in Sweden.

## The Study

HFRS has been a reportable disease in Sweden since 1989; most cases occur during fall and winter, correlating with bank vole abundance in fall (*5*,*8*–*10*). In our study, all unique HFRS cases in Sweden from July 1989 through June 2007 were grouped into HFRS seasons (July–June) and used for regression analysis and prediction. We also used reported cases in the July 2007 through June 2008 season to confirm our risk prediction.

Data on bank vole abundance in spring and fall within the HFRS-endemic region have been available since fall 1971 through monitoring by Umeå University. Collection of these data is part of the National Environmental Monitoring Programme under the administration of the Swedish Environmental Protection Agency (*11*–*13*).

**Figure 1:**
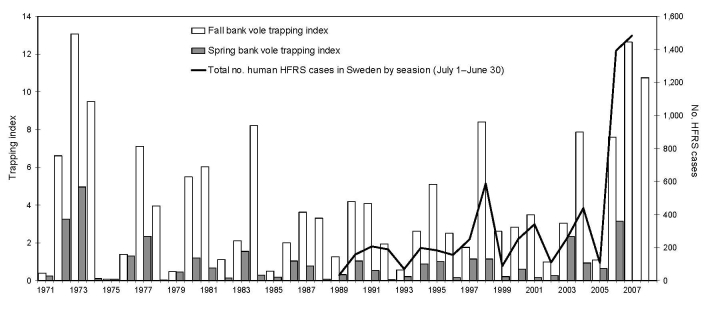
Human cases of hemorrhagic fever with renal syndrome (HFRS) by 12-month periods from July 1 through June 30 (black line), starting July 1989, when HFRS became reportable in Sweden, and ending June 2008. Bank vole trapping index in fall (white bars) and spring (gray bars) are shown from fall 1971 through 2007. Bar for fall 2007 represents predicted (*7*) trapping index. Bar on far right represents subsequently obtained actual trapping index.

The number of HFRS cases increased during the 18 twelve-month study periods but with considerable interannual variation coupled with the 3- to 4-year population cycles of the bank vole (Figure 1). Before fall sampling in 2007, we forecast bank vole abundance (*7*) by multiplying the spring index (3.16 bank voles/100 trap-nights) with the expected population growth rate from spring to fall. Previous observations showed that the growth rate decreases gradually throughout the cycle. During the 10 previous bank vole cycles studied in the same phase during fall of 2007, i.e., year 2 in the cycle, the population increased by a factor ranging from 3.4 to 13.1; median was >5 (*11*–*13*). Trapping indices of fall 2006 and spring 2007 show a striking resemblance to those in 1972–73, when the bank vole population subsequently increased 4-fold during the summer of 1973 (*11*–*13*). A 4-fold increase was thus conceived as a reasonable assumption of the growth rate during the summer of 2007, giving an estimated fall trapping index of 12.64 in 2007. A more conservative assumption of a 3-fold increase gave an index of 9.48, and a bolder estimate based on a 5-fold increase gave an index of 15.80. Thus, the fall trapping index of 2007 would most likely range from 9.48 to 15.80 bank vole specimens per 100 trap-nights, which is 20%–95% higher than 7.64, the index in fall of 2006 (*7*). The trappings showed that the fall index attained in 2007 was 10.76, i.e., ≈41% higher than in the previous year (*13*).

**Figure 2:**
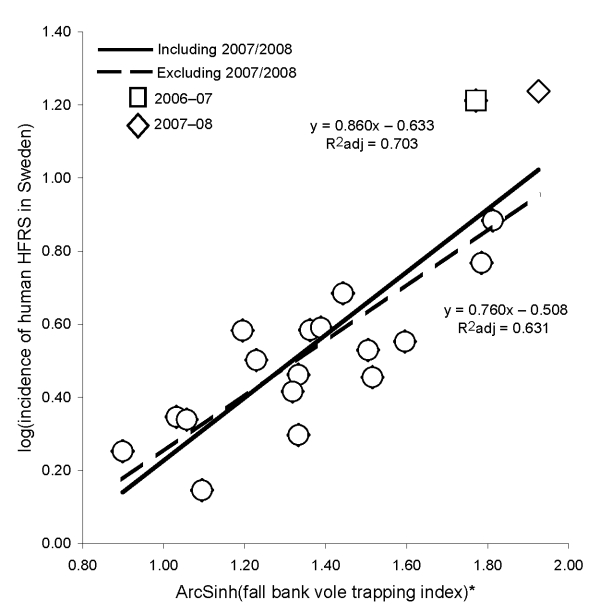
Linear regressions of incidence of human cases of hemorrhagic fever with renal syndrome in Sweden per 12-month period (July–June) from 1989 through 2007, based on fall bank vole trapping indexes from late September of the concurrent season (p< 0.001). *Because trapping indices are proportions, the arcsine transformation was used.

Linear regression analysis showed a positive correlation between incidence of HFRS cases in Sweden and fall bank vole indices (Figure 2). Using the obtained regression equation including all seasons from 1989–90 through 2006–07 and considering an observed fall index of 10.76, we estimated that the number of HFRS cases during 2007–08 would reach 1,274. Based on the a priori predicted indices of 9.48, 12.64, and 15.80, the number of HFRS cases during 2007–08 was estimated to reach 1,152, 1,451, and 1,741, respectively. The total number of reported unique HFRS cases in Sweden during this period reached 1,483 (Figure 2).

The generally higher peaks and seemingly also higher trough levels in numbers of HFRS cases in Sweden during the past decade, compared with the preceding one (Figure 1), may partly be explained by a better recognition of clinical symptoms and improved diagnostic tools. However, we believe that the increase is real, and we advance 2 hypotheses, which are not mutually exclusive, to explain the general increase of HFRS cases and the unexpected high numbers of notified HFRS cases in 2006–07 and 2007–08.

First, there seems to have been an increase of low-phase abundance and spatial distribution of bank voles and a shorter duration of the low phase in later years (*12*). These 3 factors may in turn, acting alone or in concordance, have increased the rate of PUUV transmission and increased the successive build-up of infection rates among bank voles during the population cycles relative to the situation in the early 1980s (*8*), resulting in increased rates of human encounters with infectious voles. Second, as we recently suggested (*7*), milder winters (*14*) with a less persistent and less protective snow cover, either part of normal weather variation or caused by global warming, may trigger the behavior of bank voles to more frequently leave unfavorable natural habitats and enter human dwellings for shelter. Thereby, bank vole–human encounter rates and PUUV transmission to humans would increase. Also, among HFRS patients in Sweden who were confident about time and place of PUUV exposure, >80% claimed that they were exposed inside or adjacent to a human dwelling (*5*). Thus, we suggest that a combination of high reservoir numbers (Figure 1) and a sudden extensive loss of protective snow cover likely caused the abrupt increase of HFRS cases in midwinter 2006/07 (Appendix Figure) because of the abundant infestations of human dwellings by bank voles. Under such circumstances, the virus may also circulate more successfully between aggregated conspecifics and thus further increase the risk to cohabiting humans, as observed in North America (*15*). The still elevated number of cases in 2007–08 was likely caused by excessive bank vole numbers in late 2007 but also seemed related to the loss of snow cover (in coastal areas only), which led to a less pronounced midwinter peak than in 2006–07.

## Conclusions

We found a strong positive correlation and a repeated temporal pattern between bank vole abundance and HFRS cases in Sweden. We used monitoring data to first predict and later verify bank vole abundance in fall and also to predict the risk to humans of acquiring HFRS during the fall/winter of 2007–08. Results obtained were used to raise public awareness and clinical preparedness, yet during July 2007 through June 2008, a total of 1,483 HFRS diagnoses were reported, a new high.

## Supplementary Material

Appendix Figure Human cases of hemorrhagic fever with renal syndrome (HFRS) per month from HFRS-endemic Västerbotten County, Sweden, July 2004 through June 2008, and measured snow depth at 3 locations through February 2008. The season 2004–05 represents the most recent epidemic peak year, before the large outbreak of 2006–07; 2005–06 represents an ordinary low-incidence season. The exceptional increase of HFRS cases in midwinter 2006–07 followed a rapid snowmelt and complete loss of protective snow cover to the voles during December 2006 in inland and coastal areas. Similarly, the less pronounced increase in midwinter 2007–08 followed a less abundant loss of snow cover only in the coastal area.
