# Supplementary figures and images for: Predicting High Risk for Human Hantavirus Infections, Sweden
Source: Emerg Infect Dis. 2009 Jan;15(1):104–6. doi: 10.3201/eid1501.080502 (PMC2660694; doi:10.3201/eid1501.080502)

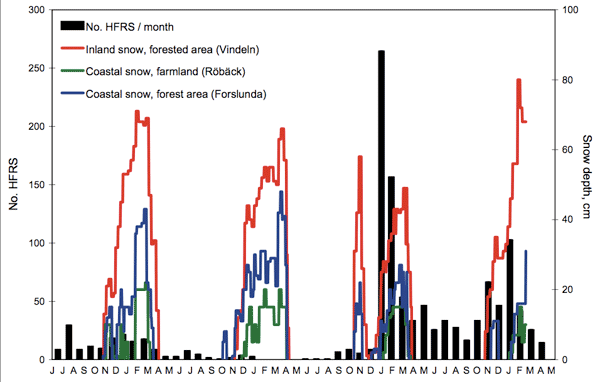

Supplement: Appendix Figure — Human cases of hemorrhagic fever with renal syndrome (HFRS) per month from HFRS-endemic Västerbotten County, Sweden, July 2004 through June 2008, and measured snow depth at 3 locations through February 2008. The season 2004–05 represents the most recent epidemic peak year, before the large outbreak of 2006–07; 2005–06 represents an ordinary low-incidence season. The exceptional increase of HFRS cases in midwinter 2006–07 followed a rapid snowmelt and complete loss of protective snow cover to the voles during December 2006 in inland and coastal areas. Similarly, the less pronounced increase in midwinter 2007–08 followed a less abundant loss of snow cover only in the coastal area. [file 08-0502_app-s1.gif]
